# Supplementary material for: Road traffic accident-related thoracic trauma: Epidemiology, injury pattern, outcome, and impact on mortality—A multicenter observational study
Source: PLoS One. 2022 May 6;17(5):e0268202. doi: 10.1371/journal.pone.0268202 (PMC9075643; doi:10.1371/journal.pone.0268202)
Supplement: Attachment — Submitted filename: response to reviewing TTF.docx [file pone.0268202.s001.docx]

We would like to thank all the reviewers for their helpful comments and suggestions, which we believe will greatly improve the quality of our work.

Please find bellow a point-by-point response to it.

Thank you very much,

Best regards,

| Journal Requirements |  |
| --- | --- |
| Please ensure that your manuscript meets PLOS ONE's style requirements, including those for file naming | This point has been addressed as requested |
| Please provide additional details regarding participant consent. In the ethics statement in the Methods and online submission information, please ensure that you have specified (1) whether consent was informed and (2) what type you obtained (for instance, written or verbal, and if verbal, how it was documented and witnessed). If your study included minors, state whether you obtained consent from parents or guardians. If the need for consent was waived by the ethics committee, please include this information.  If you are reporting a retrospective study of medical records or archived samples, please ensure that you have discussed whether all data were fully anonymized before you accessed them and/or whether the IRB or ethics committee waived the requirement for informed consent. If patients provided informed written consent to have data from their medical records used in research, please include this information | This point has been addressed as requested |
| We note that the grant information you provided in the ‘Funding Information’ and ‘Financial Disclosure’ sections do not match.  When you resubmit, please ensure that you provide the correct grant numbers for the awards you received for your study in the ‘Funding Information’ section. | This point has been addressed has requested |
| Reviewer #1 |  |
| The manuscript is organized and the prose is clearly written. Overall, the authors have done a fine job of describing the cohort and identifying trends in the data, particularly with regard to age. However, I think much more can be accomplished with the data they already have in hand. The authors need to take the element of time into consideration given the 20-year span of the dataset. Trauma care has changed substantially over the 20 years of the study. At the very least, year of injury could be included in the regression models as random parameters. One could go so far as comparing standardized survival ratios over time. However, this is completely at the authors’ discretion. Nonetheless, the matter of time needs to be acknowledged as a variable in Table 1 and included in the results, as appropriate. | Thank you for this comment. We think this is a great idea to improve our manuscript. Therefore, we have added the year of injury in the model as a continuous variable to assess whether or not it had an impact on mortality.  We also tested the year of inclusion as a categorical variable. This did not change our results. In both cases, it was not associated with mortality. This analysis is not reported in our results because we believe year of inclusion as a continuous variable is more relevant.  We also added year of inclusion in table 1 as recommended. Mortality by period of time has also been added in our results. |
| The authors give a good description of the limitations of the Rhone RTA registry. Is there information regarding chronic comorbid diseases in the Rhone RTA registry? If such data are available, inclusion in the analyses would be instructive. If not, fine. | Unfortunately, there is no such data in our registry. We have emphasized this point in the limitation section |
| How was missing data handled? Was there any missing data? Some mention of missingness would be useful to readers. | Missing data were not imputed. We added this information in the analysis (methods) section and provided number of missing data for each variable (if applicable) in table 1. As you will see, there was very few missing data. |
| The description of the logistic model fitting process is appreciated. Could the authors please include the area under the receiver operating characteristic curve to convey the discrimination of the model? | In the model, AUC was 0.9618 which is excellent. Yet, indicating the AUC is recommended in predictive modeling which is not the case herein as we propose an explanatory model   |
| The third sentence of the introduction, line 80, “Furthermore…” needs to be clarified. The phrases “third most common cause of death…” and “up to 25% of trauma-related deaths” need to be reconciled somehow. | This paragraph has been rewritten as requested |
| Reviewer #2 |  |
| The first is the inclusion of subjects prior to the adoption of whole body CT scanning. The diagnoses of rib fractures and pulmonary injuries - for example - can be considered under-reported. | We totally agree with this comment. Therefore, this limit has been well acknowledged in the discussion section (limitation)  To validate this hypothesis, we assessed the number of pulmonary contusions (which reflect the type of pulmonary injuries that were probably underreported before the adoption of WBCT) in our population over the years. We found a quasi-linear increase. Nevertheless, we do not believe this would be worthy for the reader to add this result. |
| Secondly , road crash fatalities rates had reduced by 50% in developed systems during the reporting period (1997-2018), yet the cohort is presented as a uniform population (see, for example, https://www.statista.com/statistics/437904/number-of-road-deaths-in-france/ accessed 21Feb2022). | Thank you for this comment which we believe if of great interest to improve our study.  Between 1997-2001 and 2012-2016, we noted a 38.7% decrease of patients included in the AIS≥2 group while this was only of 23.6% in the group of patients with a thoracic trauma. This information has been added in the result section. We also added year of inclusion in the logistic regression model to take the element of time into consideration as suggested as trauma patients has deeply evolved over the last decade.  Nevertheless, the objective of the study was to provide a comprehensive overview of RTA-related trauma not to assess the evolution of those trauma over the years. This very interesting point will be addressed in a dedicated future study |
| Would the authors consider: 1. Including more contemporaneous data (e.g. 1997-2021)? | 2021 data is not available. As for 2020 data, it has been highly impacted by the lockdown in France. In consequence, road accidents have drastically decreased and do not represent road accident-related thoracic injuries reality.  We do not believe that adding 2018-2019 data in our analyses would increase the quality of our findings neither would it have a significant impact on the conclusion. |
| 2. Analysing the incidence of diagnoses as well as the changes in mortality and outcomes over time? | This is indeed an excellent idea, and another study is underway to present these results (evolution of the injury pattern and outcomes of RTA-related thoracic trauma) |
