# Supplementary material for: Road traffic accident-related thoracic trauma: Epidemiology, injury pattern, outcome, and impact on mortality—A multicenter observational study
Source: PLoS One. 2022 May 6;17(5):e0268202. doi: 10.1371/journal.pone.0268202 (PMC9075643; doi:10.1371/journal.pone.0268202)
Supplement: Attachment — Submitted filename: Response to Reviewers.docx [file pone.0268202.s002.docx]

We would like to thank all the reviewers for their comments.

Only one comment has been raised on point 6 “review comments to the author: One final comment - you conclude 'Significant RTA-related thoracic injuries were rare' - but I think you mean 'Major cardiac, vascular, tracheobronchial and oesophageal injuries were rare...'?”

Thank you very much,

Best regards,

Response to the reviewer:

Thank you for this comment. This has been rephrased to be more precise. Indeed, <5% of our population sustained a moderate to lethal thoracic injury, we therefore concluded that these injuries were rare.
